# *N*-(4-Chloro­phen­yl)-4-methyl­piperazine-1-carboxamide

**DOI:** 10.1107/S1600536811034283

**Published:** 2011-08-27

**Authors:** Yu-Feng Li, Wen-Mei Wang

**Affiliations:** a Microscale Science Institute, Department of Chemistry and Chemical Engineering, Weifang University, Weifang 261061, People’s Republic of China; b Center of Forestry Science, Linjiacun Town, Zhucheng 261000, People’s Republic of China

## Abstract

In the title compound, C_12_H_16_ClN_3_O, the piperazine ring has a chair conformation. Within this ring, the *N*-methyl nitro­gen atom has a pyramidal geometry and the *N*-carboxamide nitro­gen atom is almost planar (bond-angle sum = 359.8°). In the crystal, the mol­ecules are linked by N—H⋯O hydrogen bonds into *C*(4) chains propagating in [010].

## Related literature

For related structures, see: Arrieta *et al.* (2007 ▶1); Li (2011*a* ▶,*b* ▶).
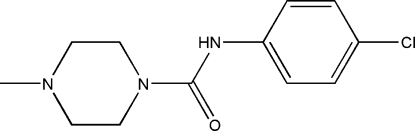


## Experimental

### 

#### Crystal data

C_12_H_16_ClN_3_O
*M* *_r_* = 253.73
Orthorhombic, 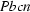

*a* = 24.920 (5) Å
*b* = 9.5033 (19) Å
*c* = 11.064 (2) Å
*V* = 2620.3 (9) Å^3^
*Z* = 8
Mo *K*α radiation
μ = 0.28 mm^−1^
*T* = 293 K
0.22 × 0.20 × 0.18 mm

#### Data collection

Bruker SMART CCD diffractometer
23785 measured reflections
3006 independent reflections
2077 reflections with *I* > 2σ(*I*)
*R*_int_ = 0.045

#### Refinement

*R*[*F* ^2^ > 2σ(*F* ^2^)] = 0.059
*wR*(*F* ^2^) = 0.192
*S* = 1.11
3006 reflections
154 parameters
H-atom parameters constrained
Δρ_max_ = 0.25 e Å^−3^
Δρ_min_ = −0.60 e Å^−3^

### 

Data collection: *SMART* (Bruker, 1997 ▶); cell refinement: *SAINT* (Bruker, 1997 ▶); data reduction: *SAINT*; program(s) used to solve structure: *SHELXS97* (Sheldrick, 2008 ▶); program(s) used to refine structure: *SHELXL97* (Sheldrick, 2008 ▶); molecular graphics: *SHELXTL* (Sheldrick, 2008 ▶); software used to prepare material for publication: *SHELXTL*.

## Supplementary Material

Crystal structure: contains datablock(s) global, I. DOI: 10.1107/S1600536811034283/hb6351sup1.cif

Structure factors: contains datablock(s) I. DOI: 10.1107/S1600536811034283/hb6351Isup2.hkl

Additional supplementary materials:  crystallographic information; 3D view; checkCIF report

## Figures and Tables

**Table 1:** Hydrogen-bond geometry (Å, °)

*D*—H⋯*A*	*D*—H	H⋯*A*	*D*⋯*A*	*D*—H⋯*A*
N3—H3*A*⋯O1^i^	0.86	2.26	3.001 (2)	144

Symmetry code: (i) 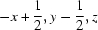.

**Fig. 1.**
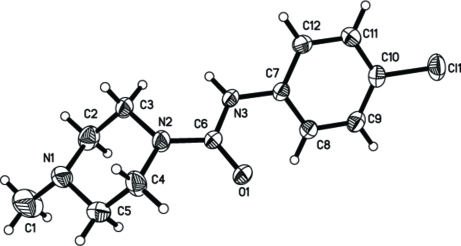
The molecular structure of the title compound showing 30% probability displacement ellipsoids.

## supplementary crystallographic information

### Experimental

A mixture of 1-methylpiperazine (0.1 mol), and (4-chlorophenyl)carbamic chloride (0.1 mol) was stirred in refluxing ethanol (20 ml) for 4 h to afford the title compound (0.079 mol, yield 79%). Colourless blocks of the title compound were obtained by recrystallization from ethanol at room temperature.

### Refinement

H atoms were fixed geometrically and allowed to ride on their attached atoms, with C—H distances = 0.93–0.97 Å; N—H = 0.86Å and with *U*_iso_(H) = 1.2*U*_eq_(C,*N*) or 1.5*U*_eq_(C_methyl_).

### Crystal data

**Table:**

C_12_H_16_ClN_3_O	*D*_x_ = 1.286 Mg m^−^^3^
*M**_r_* = 253.73	Mo *K*α radiation, λ = 0.71073 Å
Orthorhombic, *P**b**c**n*	Cell parameters from 2077 reflections
*a* = 24.920 (5) Å	θ = 3.2–27.4°
*b* = 9.5033 (19) Å	µ = 0.28 mm^−^^1^
*c* = 11.064 (2) Å	*T* = 293 K
*V* = 2620.3 (9) Å^3^	Block, colorless
*Z* = 8	0.22 × 0.20 × 0.18 mm
*F*(000) = 1072	

### Data collection

**Table:**

Bruker SMART CCD diffractometer	2077 reflections with *I* > 2σ(*I*)
Radiation source: fine-focus sealed tube	*R*_int_ = 0.045
graphite	θ_max_ = 27.5°, θ_min_ = 3.3°
φ and ω scans	*h* = −31→32
23785 measured reflections	*k* = −12→10
3006 independent reflections	*l* = −14→14

### Refinement

**Table:**

Refinement on *F*^2^	Primary atom site location: structure-invariant direct methods
Least-squares matrix: full	Secondary atom site location: difference Fourier map
*R*[*F*^2^ > 2σ(*F*^2^)] = 0.059	Hydrogen site location: inferred from neighbouring sites
*wR*(*F*^2^) = 0.192	H-atom parameters constrained
*S* = 1.11	*w* = 1/[σ^2^(*F*_o_^2^) + (0.1054*P*)^2^ + 0.5647*P*] where *P* = (*F*_o_^2^ + 2*F*_c_^2^)/3
3006 reflections	(Δ/σ)_max_ < 0.001
154 parameters	Δρ_max_ = 0.25 e Å^−^^3^
0 restraints	Δρ_min_ = −0.60 e Å^−^^3^

### Special details

**Table:**

Geometry. All e.s.d.'s (except the e.s.d. in the dihedral angle between two l.s. planes) are estimated using the full covariance matrix. The cell e.s.d.'s are taken into account individually in the estimation of e.s.d.'s in distances, angles and torsion angles; correlations between e.s.d.'s in cell parameters are only used when they are defined by crystal symmetry. An approximate (isotropic) treatment of cell e.s.d.'s is used for estimating e.s.d.'s involving l.s. planes.
Refinement. Refinement of *F*^2^ against ALL reflections. The weighted *R*-factor *wR* and goodness of fit *S* are based on *F*^2^, conventional *R*-factors *R* are based on *F*, with *F* set to zero for negative *F*^2^. The threshold expression of *F*^2^ > σ(*F*^2^) is used only for calculating *R*-factors(gt) *etc*. and is not relevant to the choice of reflections for refinement. *R*-factors based on *F*^2^ are statistically about twice as large as those based on *F*, and *R*- factors based on ALL data will be even larger.

### Fractional atomic coordinates and isotropic or equivalent isotropic displacement parameters (Å^2^)

**Table:**

	*x*	*y*	*z*	*U*_iso_*/*U*_eq_	
Cl1	0.07382 (3)	0.14060 (8)	0.66714 (8)	0.0750 (3)	
O1	0.28704 (6)	0.11160 (14)	1.01352 (17)	0.0559 (5)	
C6	0.28896 (7)	−0.0180 (2)	1.0143 (2)	0.0424 (5)	
C7	0.21110 (7)	−0.0415 (2)	0.88098 (19)	0.0399 (5)	
N3	0.25282 (6)	−0.09932 (17)	0.95154 (18)	0.0453 (5)	
H3A	0.2558	−0.1894	0.9555	0.054*	
C12	0.16012 (8)	−0.0996 (2)	0.8900 (2)	0.0479 (5)	
H12A	0.1541	−0.1754	0.9415	0.058*	
C11	0.11817 (8)	−0.0447 (3)	0.8224 (2)	0.0516 (6)	
H11A	0.0840	−0.0836	0.8282	0.062*	
C10	0.12738 (8)	0.0675 (2)	0.7469 (2)	0.0501 (5)	
C9	0.17805 (9)	0.1239 (2)	0.7340 (2)	0.0522 (6)	
H9A	0.1840	0.1981	0.6809	0.063*	
C8	0.21988 (8)	0.0687 (2)	0.8010 (2)	0.0464 (5)	
H8A	0.2542	0.1057	0.7925	0.056*	
N2	0.32650 (7)	−0.08928 (19)	1.0782 (2)	0.0558 (6)	
N1	0.43172 (7)	−0.1934 (2)	1.1165 (2)	0.0605 (6)	
C3	0.33749 (9)	−0.2391 (2)	1.0715 (3)	0.0593 (7)	
H3B	0.3139	−0.2824	1.0126	0.071*	
H3C	0.3306	−0.2822	1.1495	0.071*	
C4	0.36406 (10)	−0.0151 (3)	1.1554 (3)	0.0681 (8)	
H4A	0.3592	−0.0450	1.2385	0.082*	
H4B	0.3572	0.0852	1.1512	0.082*	
C5	0.42085 (10)	−0.0446 (3)	1.1162 (3)	0.0719 (8)	
H5A	0.4265	−0.0074	1.0356	0.086*	
H5B	0.4456	0.0025	1.1705	0.086*	
C2	0.39479 (11)	−0.2641 (3)	1.0361 (3)	0.0647 (7)	
H2A	0.4021	−0.3643	1.0372	0.078*	
H2B	0.4004	−0.2305	0.9542	0.078*	
C1	0.48742 (12)	−0.2234 (5)	1.0819 (4)	0.1134 (15)	
H1A	0.5114	−0.1767	1.1367	0.170*	
H1B	0.4937	−0.1902	1.0012	0.170*	
H1C	0.4936	−0.3231	1.0851	0.170*	

### Atomic displacement parameters (Å^2^)

**Table:**

	*U*^11^	*U*^22^	*U*^33^	*U*^12^	*U*^13^	*U*^23^
Cl1	0.0629 (4)	0.0773 (5)	0.0846 (6)	0.0211 (3)	−0.0201 (3)	0.0074 (4)
O1	0.0555 (9)	0.0286 (7)	0.0836 (13)	0.0032 (6)	−0.0119 (8)	−0.0023 (7)
C6	0.0424 (10)	0.0319 (9)	0.0529 (13)	0.0009 (7)	−0.0027 (8)	0.0008 (8)
C7	0.0433 (10)	0.0341 (9)	0.0422 (11)	0.0036 (8)	−0.0015 (8)	0.0007 (8)
N3	0.0477 (9)	0.0295 (8)	0.0587 (12)	0.0000 (7)	−0.0117 (8)	0.0055 (7)
C12	0.0493 (11)	0.0431 (11)	0.0514 (13)	−0.0051 (9)	−0.0025 (9)	0.0047 (9)
C11	0.0419 (10)	0.0536 (13)	0.0591 (15)	−0.0025 (9)	−0.0016 (9)	−0.0004 (11)
C10	0.0499 (12)	0.0490 (12)	0.0514 (13)	0.0120 (9)	−0.0052 (9)	−0.0021 (10)
C9	0.0592 (13)	0.0455 (11)	0.0520 (14)	0.0077 (9)	0.0019 (10)	0.0093 (10)
C8	0.0433 (10)	0.0436 (11)	0.0523 (13)	0.0014 (8)	0.0018 (9)	0.0060 (9)
N2	0.0542 (11)	0.0345 (9)	0.0787 (15)	0.0054 (8)	−0.0240 (10)	−0.0057 (9)
N1	0.0428 (10)	0.0548 (12)	0.0838 (16)	0.0033 (8)	−0.0014 (9)	−0.0012 (11)
C3	0.0574 (13)	0.0342 (11)	0.0862 (19)	0.0016 (9)	−0.0252 (12)	0.0045 (11)
C4	0.0611 (13)	0.0508 (14)	0.092 (2)	0.0102 (11)	−0.0304 (13)	−0.0211 (14)
C5	0.0595 (14)	0.0533 (14)	0.103 (2)	−0.0107 (11)	−0.0139 (14)	0.0008 (15)
C2	0.0767 (16)	0.0486 (13)	0.0689 (18)	0.0123 (12)	−0.0063 (13)	−0.0065 (12)
C1	0.0538 (18)	0.113 (3)	0.173 (4)	0.0106 (17)	0.023 (2)	−0.011 (3)

### Geometric parameters (Å, °)

**Table:**

Cl1—C10	1.745 (2)	N2—C3	1.452 (3)
O1—C6	1.232 (2)	N1—C5	1.440 (3)
C6—N2	1.355 (3)	N1—C2	1.446 (3)
C6—N3	1.375 (3)	N1—C1	1.468 (3)
C7—C8	1.388 (3)	C3—C2	1.500 (4)
C7—C12	1.389 (3)	C3—H3B	0.9700
C7—N3	1.411 (2)	C3—H3C	0.9700
N3—H3A	0.8600	C4—C5	1.506 (4)
C12—C11	1.388 (3)	C4—H4A	0.9700
C12—H12A	0.9300	C4—H4B	0.9700
C11—C10	1.374 (3)	C5—H5A	0.9700
C11—H11A	0.9300	C5—H5B	0.9700
C10—C9	1.379 (3)	C2—H2A	0.9700
C9—C8	1.383 (3)	C2—H2B	0.9700
C9—H9A	0.9300	C1—H1A	0.9600
C8—H8A	0.9300	C1—H1B	0.9600
N2—C4	1.450 (3)	C1—H1C	0.9600
			
O1—C6—N2	122.02 (19)	N2—C3—C2	110.4 (2)
O1—C6—N3	122.22 (18)	N2—C3—H3B	109.6
N2—C6—N3	115.75 (17)	C2—C3—H3B	109.6
C8—C7—C12	119.35 (19)	N2—C3—H3C	109.6
C8—C7—N3	122.00 (17)	C2—C3—H3C	109.6
C12—C7—N3	118.63 (18)	H3B—C3—H3C	108.1
C6—N3—C7	122.87 (16)	N2—C4—C5	110.3 (2)
C6—N3—H3A	118.6	N2—C4—H4A	109.6
C7—N3—H3A	118.6	C5—C4—H4A	109.6
C11—C12—C7	120.0 (2)	N2—C4—H4B	109.6
C11—C12—H12A	120.0	C5—C4—H4B	109.6
C7—C12—H12A	120.0	H4A—C4—H4B	108.1
C10—C11—C12	119.6 (2)	N1—C5—C4	111.0 (2)
C10—C11—H11A	120.2	N1—C5—H5A	109.4
C12—C11—H11A	120.2	C4—C5—H5A	109.4
C11—C10—C9	121.2 (2)	N1—C5—H5B	109.4
C11—C10—Cl1	119.27 (17)	C4—C5—H5B	109.4
C9—C10—Cl1	119.57 (18)	H5A—C5—H5B	108.0
C10—C9—C8	119.2 (2)	N1—C2—C3	111.8 (2)
C10—C9—H9A	120.4	N1—C2—H2A	109.3
C8—C9—H9A	120.4	C3—C2—H2A	109.3
C9—C8—C7	120.6 (2)	N1—C2—H2B	109.3
C9—C8—H8A	119.7	C3—C2—H2B	109.3
C7—C8—H8A	119.7	H2A—C2—H2B	107.9
C6—N2—C4	120.68 (18)	N1—C1—H1A	109.5
C6—N2—C3	126.47 (18)	N1—C1—H1B	109.5
C4—N2—C3	112.64 (17)	H1A—C1—H1B	109.5
C5—N1—C2	109.6 (2)	N1—C1—H1C	109.5
C5—N1—C1	111.6 (2)	H1A—C1—H1C	109.5
C2—N1—C1	110.5 (3)	H1B—C1—H1C	109.5

### Hydrogen-bond geometry (Å, °)

**Table:**

*D*—H···*A*	*D*—H	H···*A*	*D*···*A*	*D*—H···*A*
N3—H3A···O1^i^	0.86	2.26	3.001 (2)	144

Symmetry codes: (i) −*x*+1/2, *y*−1/2, *z*.

## References

[bb1] Arrieta, A., Otaegui, D., Zubia, A., *et al.* (2007). *J. Org. Chem.* **72**, 4313–4322. 10.1021/jo062672z 17506578

[bb2] Bruker (1997). *SMART* and *SAINT* Bruker AXS Inc., Madison, Wisconsin, USA.

[bb3] Li, Y.-F. (2011*a*). *Acta Cryst.* E**67**, o1796. 10.1107/S1600536811024123 PMC3152019 21837169

[bb4] Li, Y.-F. (2011*b*). *Acta Cryst.* E**67**, o1792. 10.1107/S1600536811024111 PMC3151799 21837165

[bb5] Sheldrick, G. M. (2008). *Acta Cryst.* A**64**, 112–122. 10.1107/S0108767307043930 18156677

